# Utilization of multiparametric prostate magnetic resonance imaging in clinical practice and focal therapy: report from a Delphi consensus project

**DOI:** 10.1007/s00345-016-1932-1

**Published:** 2016-09-16

**Authors:** M. J. Scheltema, K. J. Tay, A. W. Postema, D. M. de Bruin, J. Feller, J. J. Futterer, A. K. George, R. T. Gupta, F. Kahmann, C. Kastner, M. P. Laguna, S. Natarajan, S. Rais-Bahrami, A. R. Rastinehad, T. M. de Reijke, G. Salomon, N. Stone, R. van Velthoven, R. Villani, A. Villers, J. Walz, T. J. Polascik, J. J. M. C. H. de la Rosette

**Affiliations:** 1 Department of Urology, Academic Medical Center, Amsterdam, The Netherlands; 2 Department of Biomedical Engineering and Physics, Academic Medical Center, Amsterdam, The Netherlands; 3 Department of Surgery, Duke University Medical Center, Durham, NC USA; 4 Department of Radiology, Duke University Medical Center, Durham, NC USA; 5 Desert Medical Imaging, Indian Wells, CA USA; 6 Department of Radiology, Radboud University Nijmegen Medical Centre, Nijmegen, The Netherlands; 7 Urologic Oncology Branch, National Cancer Institute, National Institutes of Health, Bethesda, MD USA; 8 Urologische Praxis Dr. Henkel and Dr. Kahmann, Berlin, Germany; 9 CamPARI Prostate Cancer Clinic, Cambridge University Hospitals Trust, Cambridge, UK; 10 Department of Urology, Surgery and Bioengineering, University of California, Los Angeles, CA USA; 11 Department of Urology and Radiology, The University of Alabama at Birmingham, Birmingham, AL USA; 12 Department of Urology, Icahn School of Medicine at Mount Sinai, New York, NY USA; 13 Department of Radiology, Icahn School of Medicine at Mount Sinai, New York, NY USA; 14 Department of Radiation Oncology, Icahn School of Medicine at Mount Sinai, New York, NY USA; 15 Martini-Clinic Prostate Cancer Center, University Hospital Hamburg-Eppendorf, Hamburg, Germany; 16 Department of Urology, Institut Jules Bordet, Brussels, Belgium; 17 Department of Radiology, North Shore University Hospital, Northwell Health, NY USA; 18 Department of Urology, Lille University Medical Center, Lille, France; 19 Department of Urology, Institut Paoli-Calmettes Cancer Centre, Marseille, France

**Keywords:** Focal therapy, Prostate cancer, Consensus, Magnetic resonance imaging, Delphi

## Abstract

**Purpose:**

To codify the use of multiparametric magnetic resonance imaging (mpMRI) for the interrogation of prostate neoplasia (PCa) in clinical practice and focal therapy (FT).

**Methods:**

An international collaborative consensus project was undertaken using the Delphi method among experts in the field of PCa. An online questionnaire was presented in three consecutive rounds and modified each round based on the comments provided by the experts. Subsequently, a face-to-face meeting was held to discuss and finalize the consensus results.

**Results:**

mpMRI should be performed in patients with prior negative biopsies if clinical suspicion remains, but not instead of the PSA test, nor as a stand-alone diagnostic tool or mpMRI-targeted biopsies only. It is not recommended to use a 1.5 Tesla MRI scanner without an endorectal or pelvic phased-array coil. mpMRI should be performed following standard biopsy-based PCa diagnosis in both the planning and follow-up of FT. If a lesion is seen, MRI-TRUS fusion biopsies should be performed for FT planning. Systematic biopsies are still required for FT planning in biopsy-naïve patients and for patients with residual PCa after FT. Standard repeat biopsies should be taken during the follow-up of FT. The final decision to perform FT should be based on histopathology. However, these consensus statements may differ for expert centers versus non-expert centers.

**Conclusions:**

The mpMRI is an important tool for characterizing and targeting PCa in clinical practice and FT. Standardization of acquisition and reading should be the main priority to guarantee consistent mpMRI quality throughout the urological community.

**Electronic supplementary material:**

The online version of this article (doi:10.1007/s00345-016-1932-1) contains supplementary material, which is available to authorized users.

## Introduction

Recent technological advancements in multiparametric magnetic resonance imaging (mpMRI) have resulted in improved detection of clinically significant prostate cancer (PCa) and are increasingly used in urological practice and for focal therapy (FT). MpMRI most commonly includes T1-2-weighted imaging, dynamic contrast-enhanced (DCE) and diffusion-weighted imaging (DWI), providing clinicians with meaningful information regarding lesion volume, morphology, location and disease extent. Dynamic three-dimensional lesion characterization and risk assessment with mpMRI is key for adequate patient selection and treatment planning for FT. Clinical guidelines for standardized reporting and acquisition (e.g., PI-RADS v2 [1] or Likert scale) of prostate mpMRI are nowadays advised for both research and clinical practice. When comparing mpMRI and pathology following radical prostatectomy (sliced by use of a customized 3D mold), the positive predictive value (PPV) for the detection of PCa in the peripheral zone, central zone and overall prostate was 98, 100 and 98 %, respectively, whereas the negative predictive value (NPV) was 90 % for all mpMRI sequences [2]. In another series, the positive and negative predictive values were 86 and 85 % for lesions >0.2 mL and 77 and 95 % for lesions >0.5 mL [3]. Noteworthy, most excellent results on mpMRI PCa detection are published by expert centers where the quality of the mpMRI is assured by standardized acquisition, interpretation and image-pathology feedback. Hence, results may not be reflective to general urological practice (for an overview table on mpMRI PCa detection see [4, 5]). To illustrate, a recent systematic review of available literature on the detection of significant PCa by mpMRI showed that the NPV ranged from 63 to 98 % [6]; however, the majority of the included studies used prostate biopsies for histopathological validation.

Of interest for FT of the index lesion, the PPV of mpMRI was reported to be 82.6 % [7], whereas 80 % of all index tumors and 72 % of Gleason ≥7 tumors on whole mount pathology were identified by mpMRI [8]. Moreover, mpMRI-transrectal ultrasound (TRUS) fusion (cognitive and system-based) targeted biopsies (TB) has shown to decrease the detection of clinically insignificant PCa and increase de detection rate of clinically significant PCa with an absolute difference of 6.8 % between mpMRI-TRUS fusion TB versus TRUS-guided biopsies [9]. However, not all studies reached a statistically significant difference [9–15] (for systematic review see [9, 15]). Expert panels recommend to perform repeated mpMRI during surveillance following FT [4]. MpMRI-TRUS fusion TB has been shown to increase the detection of clinically significant PCa in the follow-up during active surveillance or during confirmatory biopsies of patients with previous negative TRUS-guided biopsies [16–18]. With increasing evidence, the position of mpMRI and MR-targeted biopsies to the accepted standard of PCa diagnostics needs to be re-evaluated. Therefore, an international multidisciplinary consensus project was initiated using the Delphi method, aiming to define the use of mpMRI and MR-targeted biopsies in clinical practice and focal therapy of PCa.

## Methods

### The Delphi method

This consensus project was executed following the Delphi method [19]. In short, a systematic literature search was conducted after which experts were selected and invited to participate. An online survey using online questionnaire software (www.SurveyMonkey.com) was constructed and presented to participants in three consecutive rounds. Only experts that completed all previous rounds were re-invited to participate in the subsequent round. After each round, the questionnaire was modified based on the comments provided by experts and aggregated results of the previous round were anonymously presented to allow the experts to re-evaluate their answers without peer-pressure. Subsequently, a face-to-face meeting was held to discuss the results of the online survey.

**Fig. 1:**
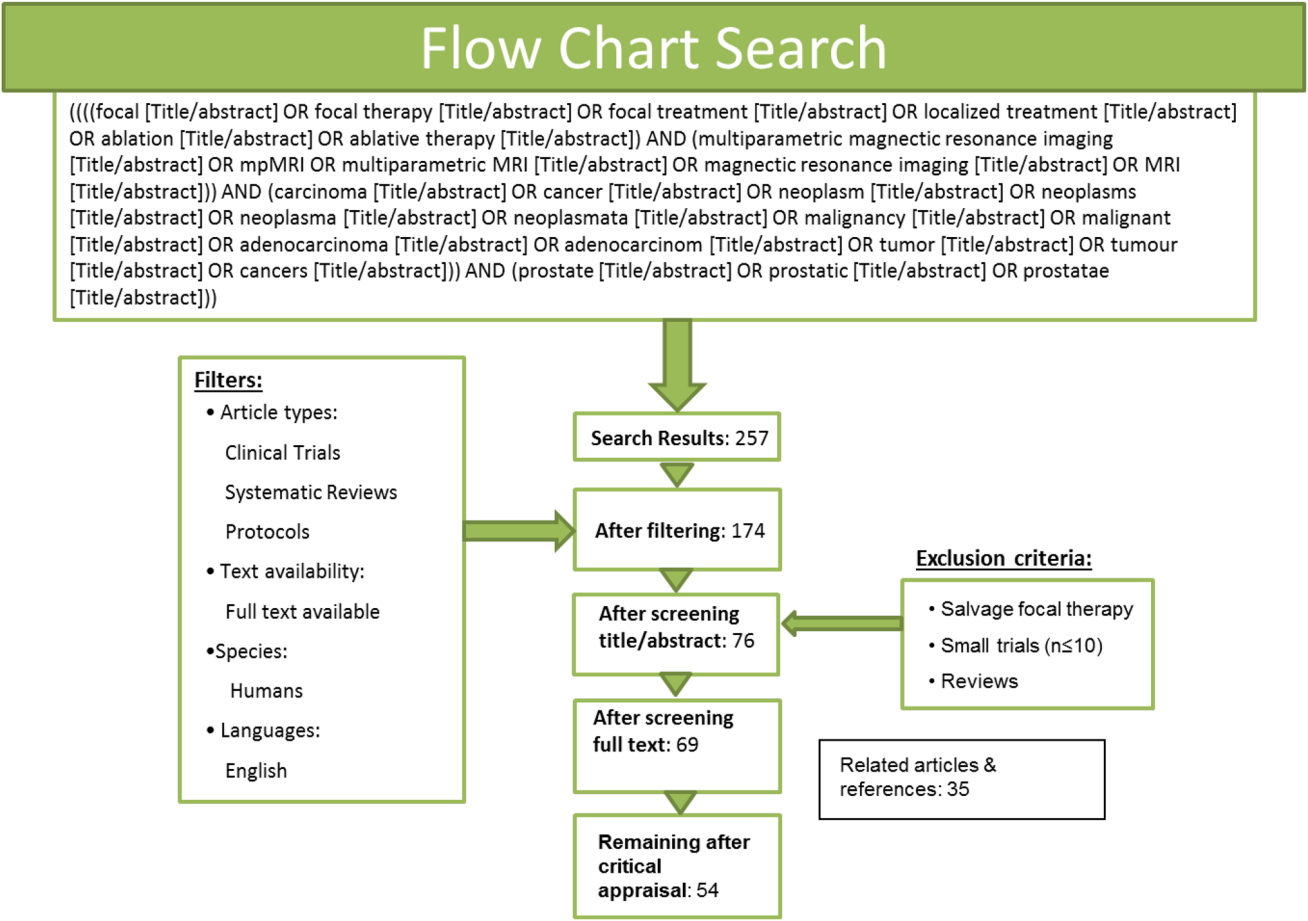
Systematic literature search

A systematic literature search of the PubMed database was performed on February 18, 2016 (see Fig. 1.), with filters: English language, full-text availability and human studies. Articles involving salvage therapy, reviews or small pilot-trials (*n* ≤ 10) were excluded. After reviewing the literature, 166 experts were invited to participate based on authorship or peer-recommendation. The online consensus process was performed between March 21 and May 27, 2016. The percentage of unanimity that should be reached to reach consensus was set at 80 %. A face-to-face panel meeting was organized on June 23, 2016, during the 9th International Symposium on Focal Therapy and Imaging in Prostate and Kidney Cancer (www.focaltherapy.org) and was attended by 17 participants (94 % completed all online rounds). Panelists discussed inconclusive results from the questionnaire in detail, as by definition results with online confirmed consensus cannot be changed. For an overview of the final results of the online questionnaire and face-to-face panel outcomes, see in ESM 1.

## Results

### Consensus process and background of participants

Ninety experts (90/166) accepted the invitation, and the response rate for the questionnaire was 100 % (90/90), 94 % (85/90), 88 % (79/90) for rounds 1, 2, and 3, respectively, and 87 % (78/90) completed all three rounds. In this group of experts 72 % were urologists, 16 % radiologists, 3 % pathologists, 3 % radiation oncologists and 6 % scientists. Average clinical experience in the field of PCa was 18.9 years (SD 9.6), 1567 years in total, and include experience with the following focal ablative modalities (primary and/or salvage); cryosurgery (*n* = 11), high-intensity focused ultrasound (*n* = 22), irreversible electroporation (*n* = 11), laser therapy (*n* = 5), vascular-targeted photodynamic therapy (*n* = 8), other (*n* = 4) and not specified/no FT (*n* = 29). Some participants had experience with more than one ablative modality for FT. A MRI-TRUS fusion system is used by 83 % (64/77) of clinicians in clinical practice to guide prostate biopsies or focal treatment procedures. A standardized mpMRI protocol (85 % PI-RADS, 7 % Likert, 2 % both PI-RADS/Likert, 2 % other) is used by 96 % (79/82) of clinicians. In conclusion, this group may provide a valid expert opinion due to the aforementioned experience of the participants with the utility of mpMRI and targeted biopsies in both clinical practice and focal therapy of PCa. For a list of all participants of the online survey and panel meeting, see in ESM 2.

### mpMRI in clinical urological practice

It is not recommended to use a 1.5 Tesla MRI scanner without an endorectal coil (ERC) or pelvic phased-array coil (81 % of whole group against, 92 % of radiologist against) and 83 % of the radiologists were also against the use of a 1.5T with a pelvic phased-array coil only. The panel emphasized that with older 1.5T systems, the use of an ERC is indispensable. When, however, newer generation 1.5T systems are used and/or if an experienced radiologist optimizes other acquisition parameters, good image quality can be obtained with 1.5T scanners without the use of an ERC. This is in line with the recommendations of the PI-RADS Steering Committee [1].

The use of mpMRI in the workup for patients suspected of PCa was codified, and consensus was reached that mpMRI should be performed after the first set of negative TRUS-guided biopsies if clinical suspicion remains (90 %), but not instead of the PSA test (96 %) nor as stand-alone diagnostic tool (95 %) or TB only (76 % with panel agreement). There was no agreement on whether or not mpMRI should be performed in the workup for all patients with suspicion of PCa, in combination with standard (10–12 core) prostate biopsies and mpMRI-TRUS fusion TB (if lesion is seen). The panel stated that only in expert centers, where the quality is assured and their own results are monitored, mpMRI may be performed in all patients suspected of PCa since it could increase the detection of clinically significant PCa, reduce the need for repeat biopsies and may avoid prostate biopsies in patients with a negative mpMRI [20]. Therefore, it could be economically justifiable, however, adequate cost-benefit studies per health care system are lacking and should be performed.

The experts were divided on whether the current NPV of mpMRI is acceptable in clinical practice to rule out significant PCa (47 % agree/49 % disagree). The panel refines that it may be applicable for expert centers with a known NPV of >90 % for clinically significant PCa, but not in community practice. In line, no consensus was reached that, after the first set of negative prostate biopsies, repeat prostate biopsies can be deferred if mpMRI does not show any suspect lesions. However, according to a recent AUA-SAR consensus statement, deferral of repeat biopsy may be considered in expert centers in case of a negative mpMRI (without a strong clinical suspicion) [21].

If prostate biopsies are performed before mpMRI, the majority recommended the minimum acceptable interval to wait before performing mpMRI to be 6 weeks (68 % with panel agreement), although it would still impair the imaging quality (97 %). Literature shows that post biopsy hemorrhage was prevalent (57 %) within 6 weeks following prostate biopsies, but did not have a detrimental effect on tumor detection or staging [22, 23].

### Focal therapy planning

MpMRI should be performed for FT planning in patients with a TRUS-guided biopsy confirmed PCa (94 %), including T1-2-weighted imaging, DCE and DWI, but without magnetic resonance spectroscopic imaging (MRSI) (83 %). MRI-TRUS fusion (either system or cognitive) is the recommended technique to perform biopsies following mpMRI (93 %) if a radiographic lesion is seen and is deemed targetable. Transperineal template mapping biopsies (TTMB) (58 % in favor with panel agreement) can also be considered, whereas TRUS-guided and in bore MR-guided biopsies are the least favorable techniques (58 and 64 % against, respectively). The panel highlighted that all techniques can be considered, depending on availability and experience, but acknowledged MRI-TRUS fusion as the optimal approach. Stand-alone MRI-targeted prostate biopsies are only sufficient for FT planning without systematic (random) biopsies in patients with previous negative TTMB (86 %), but not in biopsy-naïve patients (93 %) or in patients undergoing repeat biopsies after primary FT for low- to intermediate-risk PCa (81 %). The final decision to perform FT should be based on (targeted) histopathology and should not be based on mpMRI results/PI-RADS score (86 %) alone. Histopathological confirmation remains crucial since a recent prospective evaluation of the PI-RADS v2 found a relatively low cancer detection rate of 16, 30 and 78 % for PI-RADS score 3, 4 and 5, respectively [24].

Precise Lesion size and extension cannot be adequately assessed based with the current mpMRI quality according to the available scientific data for focal treatment planning (82 %). In a small cohort (*n* = 33), it was shown that mpMRI underestimated lesion size and boundaries. This underestimation was larger for high suspicion on imaging or higher Gleason score, however, if a simulated safety margin of 9 mm was applied, all lesions would have been treated completely [25]. The panel agreed that more data are needed to optimize treatment boundaries for FT planning.

### mpMRI during and in the follow-up of focal therapy

MRI-TRUS system fusion is considered to be the best and most practical imaging modality to guide FT procedures (e.g., needle-placement) (86 % agreement). However, the panel remarked that cognitive fusion or other modalities might be acceptable, depending on availability and experience. MpMRI should be part of the follow-up (standardized care) following focal therapy (91 %), excluding MRSI (79 %, with panel agreement). MRI-TRUS fusion biopsies should be performed following mpMRI if a lesion is seen (78 %, with panel agreement). To illustrate: in 59 patients suspected of recurrent PCa following HIFU, the likelihood of finding PCa was greater with TB on lesions seen with T2-weighted and DCE-MRI compared with systematic random biopsies [26]. However, mpMRI with MRI-TRUS fusion TB cannot serve as stand-alone follow-up modality following FT and standard repeat (random) biopsies should be taken (78 %, with panel agreement). In a paper by Shah et al. on the histological outcomes after focal HIFU and focal cryosurgery, standard repeat biopsies were positive in 25 % (98/391) and 22 % (39/175) for focal cryosurgery and focal HIFU, respectively [27]. The majority of these positive post-treatment biopsies were clinically insignificant PCa, and whether these lesions would have been identified by mpMRI remains unknown. To the best of our knowledge, there are no large series comparing MR-guided TB with standard systematic repeat biopsies on their ability to detect clinically significant PCa following FT.

## Discussion

This Delphi consensus project represents the opinion of 90 FT experts, experienced with mpMRI and MRI-TRUS fusion (TB), for all recommendations see Table 1. Despite experience, it should still be regarded as expert opinion, considered level 5 evidence, may be biased by personal enthusiasm and is potentially not reflective for community-based urologists. Nonetheless, these statements can provide clinicians guidance in areas where high-level evidence is sparse and provide a basis for standardization for clinical utilization of mpMRI and/or focal therapy that could result in improved interpretation of reported series.

**Table 1:** Overview of consensus recommendations

Recommendation	Online or panel agreement
mpMRI should be performed in patients with prior negative biopsies if clinical suspicion remains	Online
mpMRI should not be performed as stand-alone diagnostic tool or with mpMRI-targeted biopsies only	Online
mpMRI should be performed following standard biopsy-based PCa diagnosis in both the planning and follow-up of FT	Online
MRI-TRUS fusion is the recommended technique to perform biopsies following mpMRI	Online
Systematic biopsies are still required for FT planning in biopsy-naïve patients and patients with residual PCa after FT	Online
Repeat biopsies should be taken during the follow-up of FT	Online
The final decision to perform FT should be based on histopathology and not be based on mpMRI results alone	Online
Only in expert centers, where the quality is assured and own results are monitored, mpMRI may be performed in all patients suspected of PCa	Panel
Only in expert centers, deferral of repeat biopsy may be considered in case of a negative mpMRI	Panel
It should be our goal to guarantee high-quality mpMRI throughout the urological community before implementing it as standard of care	Panel

The main challenge that was repeatedly encountered was the discrepancy regarding the consensus statements that can be made for expert centers versus non-expert centers. For experienced centers, where high-quality mpMRI is obtained by standardized reporting and acquisition, experience by radiologists, and where the own data are known, some excellent results have been published, establishing the mpMRI as a reliable diagnostic tool [2, 3, 6]. In community practice, this process may be less optimal, causing heterogeneous mpMRI quality with decreased sensitivity and specificity, resulting in clinically significant PCa being missed, understaging and a high number needed to image impairing the potential cost-effectiveness [20]. The panel argued that it should be our goal to guarantee high-quality mpMRI throughout the urological community before implementing it as standard of care. Important elements to achieve this are expert training, imaging-pathology feedback and/or certification. Moreover, cost-benefit studies must be performed per health care system and the availability of MRI scanners and logistics should improve [28]. Furthermore, it may be inevitable that FT can only be performed in centers where the mpMRI quality is guaranteed, since mpMRI and MRI-TRUS fusion biopsies should be performed in both the planning and follow-up of FT.

This consensus project recommended that mpMRI should be performed in patients with prior negative biopsies if clinical suspicion for PCa remains, which is stated more firmly than the EAU guideline recommendation that mpMRI may be used to evaluate the need to perform repeat biopsies [28]. Ahmed et al. presented during the 9th International Symposium on Focal Therapy and Imaging in Prostate and Kidney Cancer the first level 1 evidence that using mpMRI as a triage test, in all patients suspected of PCa (*n* = 576), can avoid unnecessary prostate biopsies and detected more clinically significant PCa (≥Gleason 4 + 3) than systematic (random) TRUS-guided biopsies. Based on their results, these authors calculated that in the United Kingdom it was cost-effective to perform mpMRI in all patients suspected of PCa (unpublished data, [20]). This underlines the panel statement that in expert centers mpMRI may be performed in all patients suspected of PCa. The outcomes regarding mpMRI acquisition and reporting are following the recommendations by the PI-RADS Steering Committee [1] that shows the potential that the PI-RADS recommendations have to effectuate uniform mpMRI.

Previous consensus meetings differed on the need for systematic biopsies when MRI-TRUS fusion TB were performed in biopsy-naïve patients [29, 30]. Since mpMRI quality is not constant throughout the field, abandoning systematic biopsies after MRI-TRUS fusion TB for FT planning in biopsy-naïve patients is not recommended by this consensus group. In line, systematic prostate biopsies should be taken during the follow-up after FT.

## Conclusions

In expert centers mpMRI evaluation of the prostate is an established component in PCa diagnostics and this consensus project aimed to define the use of mpMRI in clinical care, especially in relation to FT. In FT, mpMRI should be used for patient selection, treatment planning or guidance, and post-treatment monitoring. Newly developed MRI-TRUS (system or cognitive) fusion is increasingly being used either during prostate biopsy procedures or during treatment planning or guidance. This consensus project was conducted during the turning point whether or not mpMRI of the prostate should be standard of care in all patients with or suspected of PCa. Standardization of acquisition and reading should be the main priority to guarantee consistent mpMRI quality throughout the field to substantiate the discrepancy between what you want to recommend and what you actually can recommend.

## Electronic supplementary material

Below is the link to the electronic supplementary material.

Supplementary material 1 (DOC 75 kb)

Supplementary material 2 (DOC 109 kb)
