# Supplementary material for: Utilization of multiparametric prostate magnetic resonance imaging in clinical practice and focal therapy: report from a Delphi consensus project
Source: World J Urol. 2016 Sep 16;35(5):695–701. doi: 10.1007/s00345-016-1932-1 (PMC5397427; doi:10.1007/s00345-016-1932-1)
Supplement: Supplementary file 1 — Supplementary material 1 (DOC 75 kb) [file 345_2016_1932_MOESM1_ESM.doc]

**Utilization of Multiparametric Prostate Magnetic Resonance Imaging in Clinical Practice and Focal Therapy: report from a Delphi consensus project.**

Scheltema MJ^1a^, Tay KJ^2a^, Postema AW^1a^, de Bruin DM^1a,b^, Feller J^3^, Futterer JJ^4^, George AK^5^, Gupta RT^2b^, Kahmann F^6^, Kastner C^7^, Laguna MP^1a^, Natarajan S^8^, Rais-Bahrami S^9^, Rastinehad AR^10a,b^, de Reijke TM^1a^, Salomon G^11^, Stone N^10a,c^, van Velthoven R^12^, Villani R^13^, Villers A^14^, Walz J^15^, Polascik TJ^2a^, de la Rosette JJMCH^1a^.

Corresponding author:

MJ Scheltema MD

Email: [m.j.scheltema@amc.uva.nl](mailto:m.j.scheltema@amc.uva.nl)

Telephone: +31 20 566 6493

Fax: +31 20 566 9585

**Electronic Supplementary Material 1.** Results of Online Rounds and Panel Statements.

| **Question** | **Response** |
| --- | --- |
| **Participant Characterization** | |
| **What is your profession?**   - Urologist - Radiologist - Pathologist - Radiation Oncologist - Physicist/Researcher | 72.2%  15.6%  3.3%  3.3%  5.6% |
| **How long have you been in practice?** 18.9 years (SD 9.6), 1567 years in total |  |
| **Are you involved in FT treatment (if so, elaborate)?** Yes (67.78%), No or Not Specified (32.22%) |  |
| **Do you use a TRUS/MRI fusion system in your hospital during daily clinical prostate cancer care?**   - Yes, during prostate biopsies (either detection or follow up biopsies) - Yes, as procedure guidance - No, only for research purposes - No, we do not have a TRUS/MRI fusion system - I do not know | 71.43%  9.52%  10.71%  7.14%  1.19% |
| **What percentage of unanimity should be reached to confirm consensus?**   - 70% - 75% - 80% - 85% - 90% - I do not know | 4.76%  20.24%  60.71%  9.52%  3.57%  1.19% |
| **What mpMRI field strength do you usually use in your hospital for treatment planning?**   - 1.5 Tesla without endorectal or pelvic phased-array coil - 1.5 Tesla with endorectal coil - 1.5 Tesla with pelvic phased-array coill - 3.0 Tesla without endorectal or pelvic phased-array coil - 3.0 Tesla with endorectal coil - 3.0 Tesla with pelvic phased-array coil - There is no significant difference | 10.0%  8.24%  5.88%  29.41%  21.18%  23.53%  4.71% |
| **Which imaging modality do you usually use in your hospital for focal therapy procedure guidance (multiple answers possible)?**   - TRUS - MRI-TRUS cognitive fusion - MRI-TRUS system fusion - In bore MR-guidance - No image guidance/transperineal template mapping biopsies - I do not know / I do not perform FT | 23.33%  32.22%  55.56%  14.44%  5.56%  6.67% |
| **Do you use a standardized reporting protocol for mpMRIs in your hospital?**   - Yes, PI-RADS (v2) /ESUR - Yes, Likert Score - Yes, Likert and PI-RADS (v2)/ESUR - Other - No | 84.6%  6.6%  2.2%  2.2%  4.4% |
| **mpMRI in Clinical Practice** | |
| **mpMRI should be performed in daily clinical practice (yes/no/I do not know)**   - Instead of the PSA test for prostate cancer evaluation (2.50% / 96.25% / 1.25%) - When elevated PSA levels are found (and/or abnormal DRE) as stand-alone diagnostic tool (5.06% / 94.94% / 0.00%) - When elevated PSA levels are found (and/or abnormal DRE) and biopsies should **only** be taken when there is visible lesion on mpMRI (17.11% / 77.32% / 6.58%) - mpMRI in the work up for all patients with elevated PSA levels (and/or abnormal DRE) in combination with standard (10-12 core) and MRI-targeted prostate biopsies (if lesion is seen) (62.82% / 33.33% / 3.85%) - After the first set of negative TRUS-guided prostate biopsies (90.12% / 8.64% / 1.23%) - Only after the second set of negative TRUS-guided prostate biopsies (do not perform mpMRI in an earlier stage of PCa work up) (22.97% / 74.32% / 2.70%) |  |
| **After the first set of NEGATIVE prostate biopsies in the initial workup for patients suspected of PCa, confirmatory prostate biopsies do NOT have to be performed if mpMRI does not show any suspicious lesions;**   - Agree - Disagree - I do not know | 59.49%  37.97%  2.53% |
| **The minimum acceptable interval between prostate biopsies and mpMRI should be;**   - No interval is needed - 1 week - 6 weeks - 8-10 weeks - ≥3 months - There is not acceptable interval, mpMRI should be performed before biopsies - I do not know | 1.27%  1.27%  68.35%  16.46%  10.13%  1.27%  1.27% |
| **mpMRI (with a minimum interval) following prostate biopsies;**   - Is still impaired - Is slightly impaired - Is not impaired | 73.08%  24.36%  2.56% |
| **The current negative predictive value of mpMRI is acceptable for daily clinical practice to rule out clinically significant prostate cancer;**   - Agree - Disagree - I do not know | 46.84%  49.37%  3.80% |
| **A standardized reporting protocol for mpMRIs should be used in clinical practice;**   - Agree - Disagree - I do not know | 100.0%  0%  0% |
| **mpMRI in Focal Therapy Planning** | |
| **What mpMRI field strength is appropriate for focal therapy treatment planning (yes/no/I do not know)?**   - 1.5 Tesla without endorectal or pelvic phased-array coil (10.67% / 81.33% / 8.00%) - 1.5 Tesla with endorectal coil (54.05% / 39.19% / 6.76%) - 1.5 Tesla with pelvic phased-array coil (20.83% / 69.44% / 9.72%) - 3.0 Tesla without endorectal or pelvic phased-array coil (54.67% / 37.33% / 8.00%) - 3.0 Tesla with endorectal coil (64.86% / 25.68% / 9.46%) - 3.0 Tesla with pelvic phased-array coil (61.84% / 23.68% / 14.47%) - There is no significant difference (4.29% / 75.71% / 20.00%) |  |
| **Current mpMRI can adequately estimate lesion size/extent for focal treatment planning;**   - Agree - Disagree (not enough data available) - I do not know | 16.46%  82.28%  1.27% |
| **Which biopsy strategy should be followed (after standard biopsies) in combination with mpMRI for treatment planning in Focal Therapy (yes/no/I don’t know)?**   - MRI-TRUS fusion biopsies (92.68% / 4.88% / 2.44%) - Transperineal template-mapping biopsies (57.89% / 35.53% / 6.58%) - TRUS-guided biopsies (36.49% / 58.11% / 5.41%) - In bore MR-guided biopsies (if considered realistic) (24.32% / 63.51% / 12.16%) - No mpMRI is necessary, only transperineal template-mapping biopsies is sufficient (9.59% / 84.93% / 5.48%) |  |
| **Stand-alone MRI-targeted biopsy is sufficient for Focal Therapy planning without systematic random biopsy in (yes/no/I don’t know);**   - Biopsy naïve patients (4.82% / 92.77% / 2.41%) - patients with a previous set of negative TRUS-guided biopsies (38.46% / 58.97% / 2.56%) - patients with a previous negative TTMB biopsy (85.71% / 13.10% / 1.19%) - patients with previous low-risk PCa on 10-12 core TRUS-guided biopsy undergoing restaging biopsy for AS or FT (38.46% / 56.41% / 5.13%) - patients with previous primary focal therapy for PCa (low- to intermediate risk) (14.10% / 80.77% / 5.13%) |  |
| **Can the decision whether to perform Focal Therapy be made solely on mpMRI results (PI-RADS v2 score)?**   - Yes, all lesions with PI-RADS (v2) ≥3 should be treated - Yes, all lesions with PI-RADS (v2) ≥4 should be treated - Yes, all lesions with PI-RADS (v2) ≥5 should be treated - No, I do not solely rely on mpMRI results/PI-RADS (v2) score. The final decision to perform focal therapy should be based on (targeted) histopathology - I do not know | 5.19%  3.90%  1.30%  85.71%  3.90% |
| **Which diagnostic tool(s) should be used for Focal Therapy planning of PCa confirmed by TRUS-guided biopsies PCa (yes/no/I don’t know)?**   - mpMRI (93.67% / 5.06% / 1.27%) - mpMRI including MRSI (6.49% / 81.82% / 11.69%) - Transperineal Template-Mapping Biopsies (56.96% / 37.97% / 5.06%) - MRI-TRUS fusion biopsies (85.37% / 10.98% / 3.66%) - In bore MR-guided biopsies (if considered realistic) (23.68% / 67.11% / 9.21%) |  |
| **mpMRI during and in the follow up of Focal Therapy** | |
| **Which imaging modality is the best for focal therapy procedure guidance in normal clinical practice (yes/no/I do not know)?**   - TRUS (12.00% / 84.00% / 4.00%) - MRI-TRUS cognitive fusion (36.84% / 57.89% / 5.26%) - MRI-TRUS system fusion (85.71% / 8.33% / 5.95%) - In bore MR-guidance (28.00% / 57.33% / 14.67%) |  |
| **What diagnostic modalities should be used as part of the standardized care in the follow up after focal therapy (yes/no/I do not know)?**   - mpMRI (91.25% / 6.25% / 2.50%) - mpMRI including MRSI (30.67% / 65.33% / 4.00%) - TRUS-guided biopsies (10-12x) (47.37% / 47.37% / 5.26%) - TTMB (40.79% / 43.42% / 15.79%) - MRI-TRUS fusion (cognitive or system) guided biopsies (78.21% / 15.38% / 6.41%) |  |
| **Is mpMRI reliable as stand-alone follow up modality after focal therapy?**   - Yes, only consider recurrence/residual disease when lesion is visible on mpMRI and targeted biopsies should performed if suspect lesion is seen - No, standard repeat biopsies should be performed - I do not know | 20.78%  77.92%  1.30% |
