# Supplementary material for: Utilization of multiparametric prostate magnetic resonance imaging in clinical practice and focal therapy: report from a Delphi consensus project
Source: World J Urol. 2016 Sep 16;35(5):695–701. doi: 10.1007/s00345-016-1932-1 (PMC5397427; doi:10.1007/s00345-016-1932-1)
Supplement: Supplementary file 2 — Supplementary material 2 (DOC 109 kb) [file 345_2016_1932_MOESM2_ESM.doc]

**Electronic Supplementary Material 2.** Registered participants to the online rounds and panel meeting

| **Name** | **Country** |
| --- | --- |
| Kovacs, Georg | Germany |
| Kroepfl | Germany |
| Kurhanewicz | USA |
| Laguna | The Netherlands |
| Lazzeri | Italy |
| Lepor, H | USA |
| Lindner | USA |
| Matin, Surena | USA |
| Matsuoka, Y | Japan |
| Miano, Roberto | Italy |
| Montironi | Italy |
| Mottet, Nicolas | France |
| Muir | United Kingdom |
| Napoli | Italy |
| Natarajan, Shyam | USA |
| Nightingale, Kathryn | USA |
| Pahernik | Germany |
| Pieters, Bradley | The Netherlands |
| Polascik, Thomas | USA |
| Rais-Bahrami, Soroush | USA |
| Rastinehad, A | USA |
| Rioja | Spain |
| Robert Villani | USA |
| Salomon, Georg | Germany |
| Sanchez-Salas, Rafael | France |
| Scheltema, Matthijs | The Netherlands |
| Schostak, Martin | Germany |
| Schwerfeld | Germany |
| Scionti, Stephan | USA |
| Sivaraman, Arjun | India |
| Stone, Nelson | USA |
| Stricker | Australia |
| Tay, Jack | USA |
| Teber, Dogu | Germany |
| Thueroff, Stefan | Germany |
| Turkbey, Boris | USA |
| Ukimura | Japan |
| v Moorselaar, Jeroen | The Netherlands |
| Valerio, Massimo | United Kingdom |
| van Velthoven, Roland | Belgium |
| Varkarakis | Greece |
| Villers, Arnaud | France |
| Walz, Jochen | France |
| Ward | USA |
| Wijkstra, Hessel | The Netherlands |

| **Name** | **Country** |
| --- | --- |
| Abreu | USA |
| Ahmed, Hashim | United Kingdom |
| Algaba | Spain |
| Amin | USA |
| Andriole | USA |
| Azzouzi | France |
| Baard, Joyce | The Netherlands |
| Baco, Eduard | Norway |
| Barrett, Eric | France |
| Baumunk, Daniel | Germany |
| Bladou, Franck | Canada |
| Bossi, Alberto | France |
| Bott | United Kingdom |
| Brausi, Maurizio | Italy |
| Casanova, Ramon-Borja | Spain |
| Choyke | USA |
| Coleman, Jonathan | USA |
| Crawford, David | USA |
| Crouzet, Sebastian | France |
| De Bruin, Martijn | The Netherlands |
| De la Rosette, Jean | The Netherlands |
| De Reijke, Theo | The Netherlands |
| Delongchamps, NB | France |
| Dickinson | United Kingdom |
| Dominguez-Escrig | Spain |
| Duddalwar | USA |
| Eggener, Scott | USA |
| Feller, John | USA |
| Frauscher | USA |
| Futterer, Jurgen | The Netherlands |
| Ganzer, Roman | The Netherlands |
| Gelet | Germany |
| George, Arvin | USA |
| Ghai, Sangeet | Canada |
| Gill | USA |
| Grummet | Australia |
| Gupta, Rajan | USA |
| Hadaschik, Boris | Germany |
| Haider | USA |
| Henkel, Thomas | Germany |
| Hohenfellner, Markus | Germany |
| Huang, Jiaoti | USA |
| Jones, Stephen | USA |
| Joniau | Belgium |
| Kastner, Christoph | United Kingdom |

| **Panel Meeting** |  |
| --- | --- |
| **Name** | **Country** |
| De Bruin, Martijn | The Netherlands |
| Feller, John | USA |
| Futterer, Jurgen | The Netherlands |
| George, Arvin | USA |
| Gupta, Rajan | USA |
| Kahmann, Frank | Germany |
| Kastner, Christoph | United Kingdom |
| Natarajan, Shyam | USA |
| Nelson Stone | USA |
| Rais-Bahrami, Soroush | USA |
| Rastinehad, A | USA |
| Robert Villani | USA |
| Salomon, Georg | Germany |
| Scheltema, Matthijs | The Netherlands |
| van Velthoven, Roland | Belgium |
| Villers, Arnaud | France |
| Walz, Jochen | France |

**Utilization of Multiparametric Prostate Magnetic Resonance Imaging in Clinical Practice and Focal Therapy: report from a Delphi consensus project.**

Scheltema MJ^1a^, Tay KJ^2a^, Postema AW^1a^, de Bruin DM^1a,b^, Feller J^3^, Futterer JJ^4^, George AK^5^, Gupta RT^2b^, Kahmann F^6^, Kastner C^7^, Laguna MP^1a^, Natarajan S^8^, Rais-Bahrami S^9^, Rastinehad AR^10a,b^, de Reijke TM^1a^, Salomon G^11^, Stone N^10a,c^, van Velthoven R^12^, Villani R^13^, Villers A^14^, Walz J^15^, Polascik TJ^2a^, de la Rosette JJMCH^1a^.

Corresponding author:

MJ Scheltema MD

Email: [m.j.scheltema@amc.uva.nl](mailto:m.j.scheltema@amc.uva.nl)

Telephone: +31 20 566 6493

Fax: +31 20 566 9585
